# Biomedical laboratory science education: standardising teaching content in resource-limited countries

**DOI:** 10.4102/ajlm.v2i1.56

**Published:** 2013-06-18

**Authors:** Wendy Arneson, Cathy Robinson, Bryan Nyary

**Affiliations:** 1 American Society for Clinical Pathology Institute, Global Outreach, USA; 2 Independent consultant

## Abstract

**Background:**

There is a worldwide shortage of qualified laboratory personnel to provide adequate testing for the detection and monitoring of diseases. In an effort to increase laboratory capacity in developing countries, new skills have been introduced into laboratory services. Curriculum revision with a focus on good laboratory practice is an important aspect of supplying entry-level graduates with the competencies needed to meet the current needs.

**Objectives:**

Gaps in application and problem-solving competencies of newly graduated laboratory personnel were discovered in Ethiopia, Tanzania and Kenya. New medical laboratory teaching content was developed in Ethiopia, Tanzania and Kenya using national instructors, tutors, and experts and consulting medical laboratory educators from the United States of America (USA).

**Method:**

Workshops were held in Ethiopia to create standardised biomedical laboratory science (BMLS) lessons based on recently-revised course objectives with an emphasis on application of skills. In Tanzania, course-module teaching guides with objectives were developed based on established competency outcomes and tasks. In Kenya, example interactive presentations and lesson plans were developed by the USA medical laboratory educators prior to the workshop to serve as resources and templates for the development of lessons within the country itself.

**Results:**

The new teaching materials were implemented and faculty, students and other stakeholders reported successful outcomes.

**Conclusions:**

These approaches to updating curricula may be helpful as biomedical laboratory schools in other countries address gaps in the competencies of entry-level graduates.

## Introduction

Laboratory capacity has been diminished in resource-limited countries, but efforts to improve capacity are ongoing at present.^1^ New technologies are being introduced into the laboratory service of developing countries, including automation, flow cytometry and molecular diagnostics.^2^ There is also a focus on developing good laboratory practices through quality systems.^3^ The American Society for Clinical Pathology (ASCP) has facilitated training-of-trainers (TOT) in-service training workshops for working laboratory professionals in the areas of haematology, biochemistry, CD4 testing and management in many developing countries.

Successful implementation and maintenance of these technologies in public health laboratories has required both in-service training and the improvement of education in medical laboratory schools in order to invest in workforce development.^4,5^ During the time of new method- and instrument roll-out, in-service training has effectively helped increase laboratory capacity to meet immediate needs, but training organisations in developing countries find that other approaches are required for maintaining the momentum achieved following any training activities.^6^ Likewise, input from laboratory supervisors and faculty of medical laboratory schools gathered at stakeholders’ meetings by the Centers for Disease Control and Prevention (CDC) and its partners revealed that new biomedical laboratory technician (BMLT) graduates do not have the knowledge or competencies needed in order to meet the needs of these new technologies in medical laboratories.^7^ Recognised experts in science curriculum revision for global health settings have advised that education for students entering healthcare settings should be focused on useful skills, knowledge and attitudes for the workforce based on job descriptions and responsibilities.^8,9^ In order to make the new skills sustainable in the laboratory setting, the USA head office of the CDC directed its partner organisations to also provide a focus on medical laboratory school education to include revision of training materials to meet new competencies.^7^ Education and technical training of individuals in vocational schools and colleges prior to entry into the workforce is termed pre-service training. This has been a major focus of the global outreach mission of the ASCP.^10,11,12^

The ASCP developed a pre-service training programme which takes place in each country over an 18 month to 24 month timeframe, involving revision of curricula and teaching materials and providing training to teaching staff of biomedical laboratory science (BMLS) schools. A successful aspect of eventually increasing laboratory capacity in developing countries is through training of students in medical laboratory schools and, specifically, through curriculum revision. Curricula were revised and aligned so that both knowledge and practical skills were taught and assessed using a variety of educational resources, based on recognised educational principles.^13^ In addition, each curriculum was written to include a focus on applications and problem-solving outcomes, rather than focusing on the recall of information.

Countries requesting assistance in building laboratory capacity, with support from USA CDC, called for technical assistance in curriculum revision in certain BMLS programmes. The ASCP and the CDC instituted curriculum revisions and development of new teaching content in several African countries, including Ethiopia, Kenya and Tanzania following their request for assistance. This process began in 2007 when the ASCP assembled a team of USA -based consultants. These consultants are USA BMLS educators with proficiency in assessment, curriculum development, and teaching techniques as well as expertise in one or more disciplines within BMLS. This consultancy team created lessons for each discipline (e.g. haematology, biochemistry, immunohematology, parasitology, microbiology, histology, cytology and management). Using a model for effective curriculum revision and implementation similar to one recommended by an internationally-recognised trainer,^8^ these consultants met in each country with BMLS educators from their respective schools and with stakeholders (including experts from national reference laboratories, national CDC staff and laboratory staff from the Ministry of Health) for on-site assessments, assistance in curriculum review and revision, and mentorship as part of the pre-service training programme.^10,14,15^

Following pedagogical training of the teaching staff and curriculum revision, the general process for developing outcomes-based teaching content was to conduct workshops in order to create lesson plans and teaching lessons. Biomedical laboratory leadership from each country selected a different lesson plan template in consideration of their own needs based on stakeholder and faculty input. L. Cuban, a recognised leader in education, has noted that there are often difficulties in implementing official curricula, which may arise as a result of personal choices or due to level of teaching experience.^16^ In order to minimise these difficulties and discrepancies in what is actually taught in the classroom, a harmonised curriculum and standardised teaching content were developed in Ethiopia, Tanzania and Kenya, so that all students graduated with equivalent knowledge and competencies. This stage of curriculum revision involved the formation of teaching materials, lectures, exercises, and hands-on activities for laboratory students to learn. One- to three-week workshops were held prior to the start of the new teaching year to provide the opportunity to develop standardised teaching content in order to meet the newly-revised learning objectives. These lesson plans were shared with participants in the workshop setting and offered an opportunity to practise teaching a new topic with evaluation and input from facilitators and stakeholders.

## Research method and design

Can lesson plans and teaching lessons developed in-country with national teaching staff and international consultants be implemented effectively in order to teach newly-revised curricula? This case study describes the outcomes of these workshops held to develop teaching content with BMLS educators from three different countries. Observations and anecdotal evidence are provided in this manuscript so as to answer this question.

In Ethiopia, Tanzania and Kenya, following needs assessments of representative laboratory schools and stakeholders’ meetings to identify the scope of work and competencies needed for entry-level graduates, the medical laboratory science curriculum was revised with input from both national BMLS educators and USA BMLS educator experts. Following approval of the curriculum, workshops were held with the same educators and USA consultant educators to develop standardised medical laboratory teaching content. Whilst differences existed in the outcomes and steps taken with the medical laboratory schools in the three African countries, there were also similarities in the overall process.

In two workshop settings, biomedical laboratory science faculty members from five universities in Ethiopia, along with the USA consultants, developed standardised lessons for each of the 26 professional medical laboratory courses in the four-year Bachelor of Science (BSc) curriculum. Interactive presentations and lesson plans, developed independently by both USA-based consultants and the in-country medical laboratory educators, served as resources to assist in developing country-specific lectures. The lesson content in each of the 26 medical laboratory courses was developed to meet current course-learning objectives with an emphasis on application of skills.

In the first workshop held in Ethiopia, faculty experts created lessons collaboratively in small groups with input from larger groups represented by the five universities. The lessons were designed to fit a 1 to 2 hour didactic teaching session or a 3 hour practical session. These lessons contained references, instructor notes, appropriate illustrations, pedagogically-interactive study questions and activities. The electronic slide-presentation lessons emphasised an application of cognitive skills. Approximately half of the teaching content was developed for the entire professional curriculum in the first workshop.

Following this workshop, the national educators continued to develop the lectures and other teaching aids for their courses. Additionally, the standardised electronic presentation lectures were reviewed and gaps were identified. The same national medical laboratory faculty and USA consultants participated in a second workshop to complete the task of developing standardised teaching content (including didactic and practical sessions for all courses) and addressing gaps in the first lesson plans identified during implementation based on anecdotal evidence from the national BMLS educators.

In Tanzania, the USA consultants met in a workshop with tutors and experts from 10 government and private medical laboratory schools to develop standardised teaching content. This process was similar to that described for Ethiopia; however, the newly-developed content was unique in that it was developed for an articulated competency-based curriculum. In competency-based curricula, modules are used rather than courses. The module content was created from various series of hierarchical outcomes following a detailed analysis of competencies required in the workplace so that specific entry-level workplace tasks could be taught and assessed. In addition, the format of developing the standardised teaching content was unique when compared with the other examples described because a detailed lesson plan in the form of a teaching-session guide was created, rather than lectures with notes.

Module teaching-session guides, based on established competency outcomes and tasks, were developed in small groups by both the faculty experts and USA educator consultants. Each teaching-session guide included, (1) the module and session title, (2) learning objectives, (3) total session time, (4) resources needed, (5) content outline, (6) more detailed content listed in order with illustrations, (7) pedagogically-interactive activities, (8) at least one assessment item and (9) references.

In Kenya, the USA consultant educators developed and delivered approximately 80 lesson plans with accompanying slide presentation lessons that addressed theory and practical skills for new technologies and topics as identified by the needs assessment. This was provided at a workshop in which the Kenyan medical laboratory lecturers from 11 campuses worked collaboratively with the USA consultants and stakeholders. In discipline-specific groups, the lesson plans were adapted to Kenyan-specific terminology and laboratory applications. This teaching content served as a resource and foundation for the lectures to be adapted as needed at any time after the workshop concluded.^15^ For Kenya, the decision to include all medical laboratory lecturers from all 11 campuses was made to facilitate consensus for use of the didactic and laboratory lesson plans when teaching the courses.^15^

## Results

The faculty spokespersons from the five universities in Ethiopia were given a short survey via email, which elicited a 20% return rate. The faculty spokespersons from six laboratory schools in Tanzania were given a short survey via email and there was a 16.7% return rate. In addition, direct observations were made by technical staff from the ASCP in seven out of eight laboratory schools. There was a 100% return rate of the direct surveys given to faculty spokespersons from the 11 campuses of the Kenyan laboratory schools.

The five universities in Ethiopia implemented standardised electronic slide presentation lectures for 26 medical laboratory professional courses. Anecdotal comments from the survey of biomedical laboratory schools’ spokespersons (SP) provided the following information regarding the use of standardised teaching lesson plans in the first year:

‘The materials become uniform throughout the country, which benefits both faculty and students.’ (SP1, Department Chair)

‘Students say they are better able to understand and learn the subject matter because the lectures and labs are more organised and more interactive.’ (SP1, Department Chair)

‘Students report that they know exactly what is expected of them and that they are better prepared for exams from the wording of the objectives.’ (SP1, Department Chair)

‘The laboratory schedule for each lecture/course helps avoid ambiguity and ensures lab space is available as needed.’ (SP1, Department Chair)

‘The only reported drawback so far comes when faculty only use the outline materials on the PowerPoint^®^ slides and do not provide supplementary information. This makes more in-depth learning difficult for students without access to textbooks or the Internet.’(SP1, Department Chair)

Eight of the 10 medical laboratory programmes in Tanzania implemented the teaching-session guides for the year-one course modules and implementation was verified by direct observation by the ASCP technical staff. Anecdotal comments from a survey of BMLS spokespersons regarding the use of the lessons during the first year were as follows:

‘The general feeling among students taking this courses is that there is good understanding, although we did not give a questionnaire to see how many had a feeling of good understanding or not good understanding.’ (SP2, Head of School)

‘We enrolled 91 students in the NTA level-four, semester one, for academic year 2010–2011, but [*the number that*] remain [*are*] 77 students (and, therefore, 14 failed to progress).’ (SP2, Head of School)

‘What we did is that we compared the [*number of students who*] failed [*with those who*] passed, [*and the*] majority passed.’ (SP2, Head of School)

Results from a survey conducted by ASCP one year post-implementation of the standardised teaching content with medical laboratory lecturers from Kenya revealed the following:

‘Faculty like using the same lesson plan template for all lectures and labs as the materials are all in the same place and easy to find and use. They also report that following the lesson plan and objectives keeps them on task. At the end of the lesson, they know they have covered all the materials related to the topic. Previously, lecturers reported they wandered around and got off-topic and weren’t sure what materials they had or had not covered.’ (SP3, Head of School)

‘Lecturers reported that they liked the fact that students on every campus are receiving the same information in each class and are all prepared similarly at graduation with regard to knowledge and skills’. (SP3, Head of School)

‘Students reported that they liked the lessons better because the lecturers stayed on topic and the material was easier to learn. Students like having objectives because objectives help them know how in-depth they need to focus on specific information and because objectives help them study for exams.’ (SP3, Head of School)

‘Students appreciate (PowerPoint^®^) handouts so they can look over the information before class. They can also follow along with the lecturer more easily and, because they aren’t consumed with taking copious notes, they are able to use critical thinking skills to ask questions about the materials during the lecture or lab class.’ (SP3, Head of School)

## Ethical considerations

This study did not involve experimentation with human subjects.

### Potential benefits and hazards

There were no risks to the subjects of this study.

### Recruitment procedures

All participants in the development and assessment of the curricula, did so as part of their work responsibilities and were invited to participate with formal letters of invitation from their Ministries of Health.

### Informed consent

No informed consent was needed or received.

### Data protection

Anecdotal data was received via email from a secured website.

## Trustworthiness

Anecdotal data in the form of comments was listed directly in the study and not altered.

### Reliability

No follow up to the results has been provided at this time.

## Discussion

Lesson plans and lessons can be developed in-country with national teaching staff and international consultants in a workshop setting and can be implemented effectively in order to teach newly-revised curricula based on this evidence from three countries and 26 laboratory schools. Needs assessments prior to these curriculum workshops indicated a lack of standardised teaching content, especially in new technologies or with quality assurance. Different formats for lesson plans can be used based on the type of curriculum and the level of expertise of the teaching staff but it is important to create formal lesson plans or teaching-session guides when implementing a new curriculum that includes many new theoretical or practical skills.

Limitations included the need for alternative methods of delivering the electronic lessons when power supplies were interrupted or when computer equipment needed to be shared amongst multiple instructors. Having printers to print out lessons bridged some gaps when instructors had to revert to the blackboard or whiteboard to deliver visual aspects of the lessons. In addition, not all faculty members were able to attend the teaching content development workshops and, consequently, other teaching staff may have developed lessons for them. If proper mentoring was not provided for the use of these lesson plans, the new lesson plans became a limiting step for novice faculty members. Turnover in teaching staff sometimes accelerated this limitation. Another limitation was in planning realistic lesson plans for hands-on practice that matched the current or expected available resources. Sometimes the new curriculum called for teaching practical skills that were not fully resourced as hands-on lessons. Lesson plans that contained the critical-thinking skills but had simulations of missing resources were developed in the workshops in order to bridge gaps with respect to immediate resource limitations.

Lessons learned regarding the implementation of new content to meet the revised BMLS curriculum include the following main points:

- It is critical to the overall success of pre-service activities to conduct a pre-assessment of the university’s resources and inventory in order to circumvent challenges when the implementation of teaching content begins. For example, if lessons are developed in PowerPoint^®^, the sustainability of the resources and the lecturers’ skills must be maintained or the lessons will not be possible to implement effectively.
- Prior to the development and implementation of teaching content, it is important to the success of the pre-service programme to provide teaching-methods training to the instructors, including interactive teaching strategies, objective-writing practice, and effective practices in the development and use of electronic teaching media (such as PowerPoint^®^ and Word^®^). An emphasis on the mechanics of good visual appearance and animation was relevant.
- It was found to be important to plan for periodic meetings and reviews between faculty, campuses and universities after implementation in order to build a support system for sustainability of the curriculum and retention of the faculty.

Similarities in developing teaching content for these three African countries included the following:

- Teaching content was developed to address a standard set of medical laboratory competencies in all three countries.
- This was accomplished by a systematic approach to identifying the competencies and by including both stakeholders and USA educator consultants.
- In all three countries, the common elements of teaching content and standard format were determined in large-group settings; specific discipline content was developed in small-group settings.
- All three countries developed standardised teaching content for use in BMLS programmes throughout their country.
- All three countries implemented the standardised teaching content for the new curriculum successfully.

Differences in the implementation of content in these three countries included the following:

- Teaching content for Tanzania was developed as teaching-session guides and organised in teaching guides for competency-based modules, whilst teaching content used in Ethiopia and Kenya was developed in lesson plans and electronic presentation format and organised within the framework of traditional courses.
- Teaching content for Tanzania was organised in a prescriptive teaching guide with nine mandatory components, as opposed to teaching content organisation in Ethiopia and Kenya, which was less prescriptive, allowing for more flexibility in delivery style.
- Standardised teaching content for Ethiopia and Tanzania was completed with direct USA consultant involvement in a workshop setting, whilst the standardised teaching content for Kenya was completed by all campus lecturers following the workshop without direct involvement from the USA consultants.

## Limitations of the study

Due to the limitations of funding for the study, stakeholders have not been surveyed to determine if the newly-revised and implemented curricula meet the gaps in competencies that previous curricula did not fill. The number of new graduates trained with this new curricula have been quantified.

### Recommendations

In order to ascertain whether the new curricula provide the necessary competencies to meet the needs of each country and increase laboratory capacity in quantity and quality, surveys of employers and other stakeholders in these countries should be implemented, analysed and reported as part of future research studies.

## Conclusion

Teaching content should be revised to match the needs of the workforce and can effectively be developed by collaboration of national BMLS instructors with international BMLS educator consultants. New teaching methods, including the use of electronic slide presentations, can be used to implement content that will address new skills and emerging technologies. Providing new technologies and methods for teaching content, however, should not eliminate the need for interactive strategies to involve students in active learning. In other words, the immediate appeal of animation and images that can be provided by electronic slide presentations should be used to enhance active (rather than passive) learning. The implementation of curriculum teaching content must be in conjunction with review and revision of assessments in order to ascertain that they address directly the knowledge, skills and attitude objectives specified in each course or module.

The authors anticipate that by sharing this information, it may be helpful to biomedical laboratory faculty and administrators in other countries as they consider how to revise and implement standardised curriculum content.
